# Effects of probiotic and magnesium co-supplementation on mood, cognition, intestinal barrier function and inflammation in individuals with obesity and depressed mood: A randomized, double-blind placebo-controlled clinical trial

**DOI:** 10.3389/fnut.2022.1018357

**Published:** 2022-09-28

**Authors:** Sepideh Mahboobi, Marzieh Ghasvarian, Haleh Ghaem, Hamzeh Alipour, Shohreh Alipour, Mohammad Hassan Eftekhari

**Affiliations:** ^1^Student Research Committee, Shiraz University of Medical Sciences, Shiraz, Iran; ^2^Department of Epidemiology, Non-communicable Diseases Research Center, School of Health, Shiraz University of Medical Sciences, Shiraz, Iran; ^3^Department of Vector Biology and Control of Diseases, Research Center for Health Sciences, Institute of Health, School of Health, Shiraz University of Medical Sciences, Shiraz, Iran; ^4^Department of Pharmaceutics, Department of Pharmaceutical Quality Control, School of Pharmacy, Shiraz University of Medical Sciences, Shiraz, Iran; ^5^Department of Clinical Nutrition, School of Nutrition and Food Sciences, Shiraz University of Medical Sciences, Shiraz, Iran

**Keywords:** obesity, mood, intestinal integrity, inflammation, cognition, probiotics

## Abstract

**Background:**

The co-occurrence of obesity and mood impairments named as “metabolic mood syndrome” (MMS) is often neglected in the obesity management. This study aimed to evaluate effects of Probio-Tec ^®^BG-VCap-6.5 and magnesium co-supplementation on mood, cognition, intestinal barrier function and serum C reactive protein (CRP) levels in participants with obesity and depressed mood.

**Design:**

Seventy-four eligible participants were randomly allocated to either Probio-Tec^®^BG-VCap-6.5 [containing *Lactobacillus rhamnosus* (LGG^®^) and *Bifidobacterium animalis* subsp. *Lactis* (BB-12^®^)] + Magnesium chloride or placebo for 9 weeks. Sociodemographic data were collected in the beginning. Anthropometric, dietary and physical activity (PA) assessments were carried out. Beck Depression Inventory-II (BDI-II) and Montreal Cognitive Assessment (MoCA) scores were assessed through validated questionnaires. Fasting plasma zonulin, lipopolysaccharide (LPS) and (CRP) were measured by ELIZA kits.

**Results:**

Of seventy-four participants (mean age 37.51 ± 8.10), 52 completed the study. Changes in serum LPS and zonulin were not different significantly between groups (−3.04 ± 44.75 ng/dl, 0.11 ± 5.13, ng/dl, *p* > 0.05 for LPS and 1.40 ± 48.78 ng/dl, −0.17 ± 6.60, *p* > 0.05 for zonulin, respectively). CRP levels reduced significantly in intervention group compared to placebo [−474.75 (−1,300.00, −125.00) mg/l vs. 175.20 (−957.75, 1,683.25) mg/l, *p* = 0.016]. Changes in BDI-II and MoCA scores were not significantly different between intervention (−7.13 ± 5.67, 1.20 ± 2.16, respectively) and placebo (−5.42 ± 6.71, 1.94 ± 1.86, respectively) groups (*p* > 0.05).

**Conclusion:**

Nine weeks of probiotic and magnesium co-supplementation resulted in decreased CRP levels as an indicator of inflammatory state with no significant effects on mood, cognition and intestinal integrity in individuals with obesity and depressed mood.

## Introduction

Obesity, characterized by excessive body fat accumulation (1), is one of the most important features of metabolic syndrome (METs) associated with multiple comorbidities contributing to a lower life expectancy (2, 3). Obesity was responsible for 120 million disability-adjusted life years (DALYs), equal to 4.9% of all DALYs in 2015 (4).

According to World Health Organization (WHO), the worldwide prevalence of overweight and obesity was 39 and 13%, respectively (5). Obesity prevalence was estimated to be 22.7% in the Iranian population (6).

Research has revealed that obesity is not just a simple imbalance between calorie intake and expenditure, but a more complex neurobiological condition manifesting anxiety, depression, binge eating, and mild cognitive impairment (7, 8). A bidirectional relationship has been shown between obesity and neuropsychiatric status (9), which constitutes an illness subtype named “metabolic-mood-syndrome” (MMS) with distinct pathophysiological mechanisms, different clinical manifestation and treatment response compared to each condition, separately (10–12).

Mood disorders manifest several pathological features most of which overlap with obesity, making them powerful candidates for the etiology of MMS. Gut dysbiosis, impaired intestinal permeability, cytokine imbalances and chronic low grade systemic inflammation can be regarded as some important key players in the etiology of MMS (13–15).

The human gastrointestinal (GI) tract is resided by a large microbial community named as gut microbiota (16). Metagenomic analysis of this microbial population has revealed that intestinal microbiota can act as a metabolic organ with a variety of physiological functions including immune modulation and metabolic function (17). Several studies has reported an association between obesity and changes in both composition and function of gut microbiota including an increase in opportunistic pathogens, reduced short chain fatty acid (SCFA) producer genera and increased capacity to harvest energy from diet (18). These alterations namely gut dysbiosis (19) can impair gut physiology and disrupt intestinal barrier integrity (20–22). Gut dysbiosis is directly associated with obesity (19) and can negatively impact gut physiology and disrupt intestinal barrier integrity (20–22). Impaired intestinal permeability leads to elevated circulating bacterial derived Lipopolysaccharides (LPS) which activates Toll Like Receptor-4 (TLR-4) located on the surface of macrophages (23–25) which in turn triggers systemic and neuro-inflammation (22). Zonulin reversibly regulates intestinal tight junction proteins (occludin and zonula occludens-1) (26, 27) and is strongly correlated with the lactulose: mannitol ratio (28) which makes it a useful marker of intestinal permeability (26).

Modern world, including developing countries, has experienced a shift to more consumption of high calorie, high fat westernized diets (29, 30) which not only impair gut microbial diversity (19), but also lead to inadequate intake of micronutrients, such as vitamin B-6, magnesium, calcium and zinc, in the long run (31, 32). Magnesium is an essential micronutrient with a variety of functions in metabolism, neurotransmission and immunomodulation (33, 34). Magnesium deficiency can contribute to systemic and neuro-inflammation and involves in the pathogenesis of metabolic and psychiatric disorders (34–36). New studies are indicative of a direct association between gut microbiota and the variations in dietary magnesium intake. Some animal studies have shown that magnesium administration can enhance SCFA concentrations and gut microbiota diversity (37, 38). Magnesium deficient diet on the other hand, resulted in decreased gut *Bifidobacterium*, lower mRNA levels of tight junction proteins, as well as increased levels of Tumor Necrosis Factor-α (TNF-α) and IL-6 (39). Inadequate magnesium intake is also associated with elevated CRP levels, a common indicator of inflammatory state (40). A very recent review focused on studies in the last 3 years, has reported the possibility of adding magnesium orotate and probiotic as an adjunct treatment in individuals suffering from both GI and psychiatric disorders focusing on their ability to modulate gut-brain axis (37).

Probiotics are beneficial micro-organisms that can improve their host's health through restoration of gut microbial communities, improving intestinal barrier integrity and immunomodulation (41, 42) and suppression of body weight gain (43). Some probiotics can positively impact mental health and alleviate depressive symptoms, which are specifically called psychobiotics (44, 45). Based on evidence, several pathways can be hypothesized through which probiotics exert beneficial effects on gut-brain axis making them capable of alleviating MMS; SCFAs as a major metabolite of probiotics, participate in anti-inflammatory processes leading to increased production of IL-8 and improved gut barrier tightness (44). These SCFAs can also exert anti-obesity functions by increasing insulin sensitivity and fatty acid oxidation and decreasing fat accumulation, through the activation of AMP kinase (Adenosine Monophosphate activated Kinase) in muscles (46). Probiotic supplements suppress the expression of pro-inflammatory cytokines such as IL-6 and IL-17, and promote the expression of tight junction proteins (Zo-1, claudin-1, and occludin) (47).

Species from *Bifidobacteria* and *Lactobacilli* genera have gained the most interest in probiotic and psychobiotic- related studies (48, 49).

Studies regarding effects of probiotics on weight management, inflammation, intestinal permeability, depression and cognition, have reported inconsistent findings (42, 44, 45, 50–53). Although many clinical trials exist in the field of probiotics and obesity, to our knowledge no clinical study has targeted psychology and gut brain axis for probiotic interventions in MMS management. The anti-depressant role of magnesium has been well established in previous research (54). However, clinical studies investigating its role in improving gut barrier function is scarce. Since both magnesium and probiotics have the ability to improve gut-brain axis, we assumed their combination might exert more beneficial effects than each intervention, separately.

Gathering all these evidence and assumptions together, we designed a clinical trial evaluating effects of probiotic and magnesium co-supplementation on some parameters related to gut-brain axis in individuals with MMS.

## Subjects, material, and methods

### Sample size determination

A sample size of 60 (30 per group) was calculated based on a previous study by Steenburgen et al. (55), considering BDI-II as main variable, type I error 0.05 and type II error of 0.20 (power 80%). With a predicted attrition rate of 20%, the sample size was increased to 72 (36 per group).

### Participants, randomization, and procedures

Seventy-four men and women with obesity and depressed mood participated in this 9-week, double-blind, placebo controlled randomized clinical trial. Through local advertisements and social media, recruitment was conducted consecutively from October 2020 to February 2021, in Nutrition clinic, Imam Reza Hospital, affiliated with Shiraz University of Medical Sciences, Shiraz, Iran. In an initial screening phase, volunteers were evaluated for eligibility based on the following criteria: age 18–50 years, body mass index (BMI) ≥ 30 kg/m^2^, waist circumference (WC) ≥ 88 cm for women and 102 cm for men, and BDI-II scores between 14 and 28 (with the approval of a clinical psychiatrist). Further criteria were as follows: being non-smoker, non-alcohol/opioid addict, not suffering from any chronic condition (renal/liver/gastrointestinal/lung diseases, diabetes, severe neuropsychological or mental disorders and infections), not having a history of stroke, not taking anti-depressants, anti-inflammatory drugs and corticosteroids and not being in pregnancy, lactation and menopause states. Furthermore, participants must have not taken antibiotics, probiotic, magnesium and omega 3 supplements, at least 1 month prior to the study commencement. Because of COVID-19 pandemic, confirmed cases of COVID-19 and those with any usual symptoms of COVID-19 were not included in the study. Exclusion criteria were: any changes in usual diet, medication and physical activity, starting antibiotic therapy, the occurrence of any side effects which would stop by discontinuation of intervention and non-compliance to research instructions. The study protocol was drafted and conducted according to Declaration of Helsinki (56) and CONSORT statements (57), registered in Iranian Registry of Clinical Trials (IRCT ID: IRCT20191127045525N1) and approved by the ethics committee of Shiraz University of Medical Sciences (approval code:IR.SUMS.REC.1398.1375). After providing a written informed consent, subjects started a 2-week run- in period. In this phase, for ethical reasons, all participants were first given a general consultation for lifestyle improvement and were asked to keep their diet, physical activity and usual medications constant during run-in and intervention periods. No weight loss or specific diets were provided. Participants were also asked to avoid taking probiotic products, magnesium and omega3, as well as any anti-inflammatory or pain relieving medications during the study.

After an overnight fast and providing blood samples, participants were assessed for demographic characteristics, diet, physical activity, anthropometric parameters and cognition, and were then randomly allocated into either intervention (*n* = 39) or placebo (*n* = 35) groups through block randomization with blocks of four. Allocation order was concealed from research executors by sealed opaque envelopes containing A or B, by a third party who was not actively involved in recruitment process. Subjects in the intervention group received two separate Probio-Tec^®^BG-VCap-6.5 and magnesium chloride capsules (one capsule each) while those in placebo group received two placebo capsules for 9 weeks, on a daily basis. Subjects were instructed to store the capsules in refrigerator and take one of each after main meal. Products and compliance checklists were distributed in the start and middle (forth week) of the study. Participants were asked to record any adverse events and were in every-day contact with a trained executor through phone calls and text messages to keep compliance and discuss any probable questions. After 9 weeks of intervention a final visit was arranged to obtain post- intervention fasting blood samples, psychological and cognitive assessments, evaluating compliance and gathering dietary and physical activity data.

### Study products and blinding

Probiotics (Probio-Tec^®^BG-VCap-6.5) were a research fund received from Chr. Hansen company (Copenhagen, Demark) and contained *Lactobacillus rhamnosus* (LGG^®^) and *Bifidobacterium animalis* subsp. *Lactis* (BB-12^®^) in a ratio of 1:1 with a potency of 1.8 × 10^10^ CFU (Colony Forming Unit) per cap. Magnesium chloride powder was purchased (Pharmbio Inc., Korea), processed and capsulated in laboratory of pharmacy department, Shiraz University of Medical Sciences, Shiraz, Iran. Each capsule contained 500 mg magnesium chloride which provided 125 mg elemental magnesium (~31 and 41% RDA for women and men, respectively). While the original protocol was to provide 250 mg elemental magnesium, which needed participants to take magnesium capsules twice per day, for better compliance we decided to provide 125 mg elemental magnesium once per day. Placebos contained maltodextrin and were similar in shape, color, weight and packaging to either probiotic or magnesium chloride capsules. Therefore, neither participants nor research executors were capable of distinguishing active products vs. placebos until the analyses were completed.

### Demographic, dietary, and physical activity assessment

Data regarding general demographic, medical history and socioeconomic factors were gathered using a questionnaire designed by research team. For dietary assessments, participants filled three 24-h food records (two weekdays and one weekend day) in the start and in the last week of study period. Daily calorie and nutrient intakes were then calculated by Nutritionist IV software (First Databank, San Bruno, CA, USA) using Iranian food composition database.

Three 24-h physical activity (PA) dairies were completed by participants before and at the end of study duration (58). PA was then calculated as metabolic equivalents in hour per day (METs-hrs/day). To compute METs for each activity we calculated daily hours a person had spent on that specific activity. MET-hrs of all daily activities were then summed to calculate daily physical activity.

### Anthropometric assessment

Body weight was measured with 100 g precision using a Seca scale (Seca, Germany) while subjects were in light clothing and barefoot. Height was measured with 0.1 cm precision using wall mountable height rod on a flat surface with barefoot. The narrowest part between lowest rib and iliac crest was marked for measuring WC with an un-stretchable tape with 0.1 cm precision. BMI was calculated by dividing weight (kg) to height squared (m^2^).

### Psychologic and cognitive assessments

For mood assessment we used Beck Depression Inventory II (BDI-II), a 21-item self-administered questionnaire. For each item participants were instructed to choose the best option that described their mood during the last 2 weeks. Options of each item are rated from 0 to 3 based on symptom severity and the final score is a sum of all scores ranging from 0 to 63. Scores between 14 and 28 are indicative of mild to moderate depression. BDI-II is the most commonly used instrument for screening of depression in general population. It has a high internal consistency, reliability and structural validity and has shown the capacity to discriminate between depressed and non-depressed subjects and can be applicable for research and clinical practice worldwide (59–61). The reliability and validity of Persian BDI-II was confirmed in previous studies (60). Cognition was evaluated by Montreal Cognitive Assessment (MoCA) tool. The scoring is based on Visuospatial and executive functioning (5 points), animal naming (3 points), attention (6 points), language (3 points), abstraction (2 points), delayed recall (5 points), orientation (6 points) plus 1 extra point for those who have < 12 years of formal education. Persian MoCA is validated by Z. Nasreddin and is available on www.mocatest.org.

### Biochemical analyses

After an overnight fast (10 h), 5 cc blood samples were collected between 07:30 to 9:30 a.m. Samples were then centrifuged at 3,000 ×, serum was separated and stored at −70°C till analysis. Serum Zonulin and LPS were analyzed by Enzyme-linked Immunosorbent Assay (ELIZA) kits (both: Shanghai Crystal Day Biotech Co., China) following the instruction manual. Serum CRP was analyzed by ELIZA kit (LDN, Nordhorn, Germany) according to the manufacturer's instruction. Serum magnesium was measured by a commercially available kit (ZistChem Diagnostics, Tehran, Iran) using colorimetric method with autoanalyzer.

### Statistical analysis

Data was analyzed using SPSS software (ver.17, for windows, SPSS Inc., Chicago, USA). Normal distribution of quantitative variables was assessed using Shapiro-Wilk test as well as normality curves. Mean ± SD and median (Q1, Q3) were used to present normally and non-normally distributed variables, respectively. Categorical variables were presented as numbers and percentages. To calculate missing data for dropouts, imputation technique was carried out using mean differences obtained from existing data. In case of normal distribution, Within-group and between group comparisons were conducted using paired sample *t*-test and independent sample *t*-test, respectively. For skewed variables we applied their equivalent non-parametric tests including Wilcoxon signed ranked test and Mann-Whitney U test. Categorical variables were compared between groups by applying chi-2 test. For all tests, *p*-value ≤ 0.05 was considered significant.

## Results

**Figure 1:**
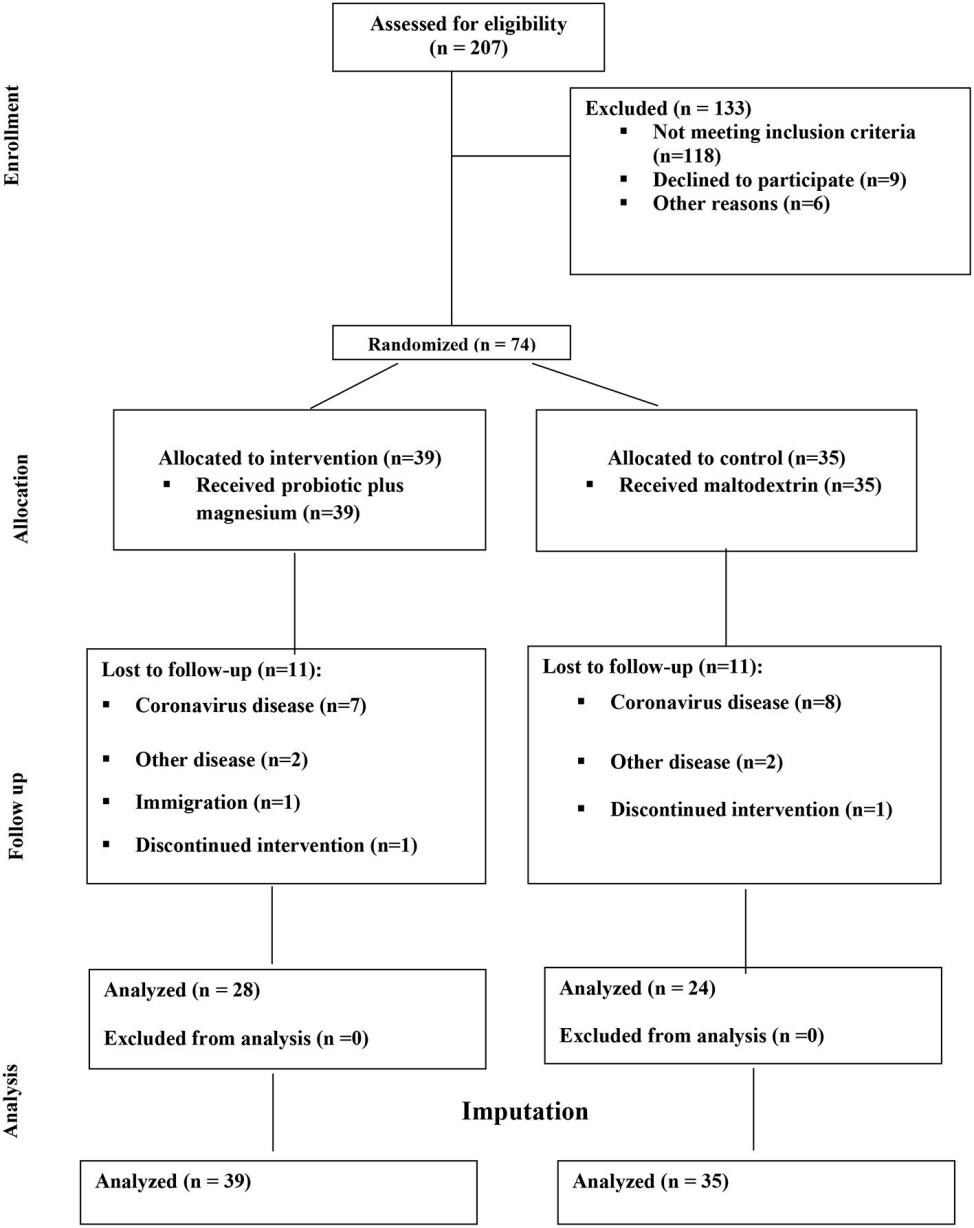
Study CONSORT flowchart.

Of 207 volunteers, 74 eligible subjects (58 women and 16 men) were randomized to either intervention or placebo groups. Fifty- two participants completed the study and were included in the final analysis. However, due to dropouts, data for missing values were computed based on imputation method explained in the previous section. Figure 1 demonstrates the study CONSORT flowchart. Mean ± SD age, BMI and BDI-II scores of study participants were 37.51 ± 8.10, 34.42 ± 3.60, 21.95 ± 7.77, respectively. Participants' baseline characteristics are presented in Table 1 compared by study group. As shown in the table, no significant differences exist between study groups in terms of age, sex BMI, WC, BDI-II, serum magnesium and sociodemographic factors at the baseline which is indicative of appropriate randomization process. Table 2 demonstrates data on calorie and nutrient intakes as well as PA of participants in the beginning and after 9 weeks. No significant within-group and between group changes were observed regarding calorie, macronutrient and micronutrient intake as well as PA, during the study. Effects of probiotic and magnesium co-supplementation on study outcomes are shown in Table 3. Serum levels of LPS and Zonulin did not significantly change in intervention (−3.04 ± 44.75 ng/dl, *p* > 0.05; 0.11 ± 5.13 ng/dl, *p* > 0.05, respectively) or placebo (1.40 ± 48.78 ng/dl, *p* > 0.05; −0.17 ± 6.60, *p* > 0.05, respectively) groups during the study. Between-group differences were also non-significant (*p* > 0.05). Our intervention resulted in reduction in serum CRP levels [−0.047 (−0.13, −0.012) mg/l] which was significantly different from its change in placebo group [0.017 (−0.095, 0.160) mg/l] (*p* = 0.016). BDI-II and MoCA scores significantly improved in both intervention (−7.13 ± 5.67, *p* < 0.001; 1.20 ± 2.16, *p* = 0.001, respectively) and placebo (−5.42 ± 6.71, *p* < 0.001; 1.94 ± 1.86, *p* < 0.001, respectively). However, between- group differences for these two outcomes were non-significant. Serum magnesium was also measured as a secondary outcome which did not significantly change post-intervention in either groups (−0.03 ± 0.16, *p* > 0.05 for intervention and 0.03 ± 0.13 for placebo group). Furthermore, these changes were not significantly different between groups.

**Table 1:** Baseline characteristics of study participants.

**Variable**	**Study groups**		***p*-value**
	**Group A^a^**	**Group B^b^**	
Sex (*n*, %)			0.411^**§**^
Male	10, 25.6	6, 17.1	
Female	29, 74.4	29, 82.9	
Age (year)	38.94 ± 7.19	35.90 ± 8.64	0.108^¶^
Education (*n*, %)			0.541^**§**^
≤ 6 years of official education	3, 7.7	6, 17.1	
6–10 years of official education	18, 46.2	17, 48.6	
B.Sc. degree	14, 35.9	10, 28.6	
M.Sc. degree and above	4, 10.3	2, 5.	
Weight (Kg)	96.92 ± 17.27	91.20 ± 13.73	0.121^¶^
BMI (kg/m^2^)	34.59 ± 3.97	34.24 ± 3.16	0.685^¶^
WC (cm)	115.42 ± 10.13	113.39 ± 8.38	0.337^¶^
BDI-II	21 (15, 29)	20.50 (15.00, 25.25)	0.685^R^
MoCA	24.92 ± 2.99	24.08 ± 3.49	0.271^¶^
Serum magnesium (mg/dl)	2.00 ± 0.16	1.97 ± 0.22	0.879^¶^
Serum LPS (ng/ml)	217.00 (179.50, 256.50)	217.00 (181.00, 264.00)	0.860^R^
Serum zonulin (ng/ml)	13.73 (8.92, 18.88)	13.02 (10.00, 18.25)	0.808^R^
Serum CRP (ng/ml)	5705.94 ± 3583.72	6201.82 ± 4705.63	0.899

^a^Group A: intervention group, received one probiotic capsule (Probio-Tec^®^BG-VCap-6.5, containing 1.8 × 10^10^ CFU *Lactobacillus rhamnosus* and *Bifidobacterium animalis* subsp. *Lactis*) plus one magnesium chloride capsule (containing 125 mg elemental magnesium), on a daily basis for 9 weeks.

^b^Group B: received two placebo capsules containing maltodextrin on a daily basis for 9 weeks.

^**§**^ *P*-values obtained from Chi-2 test.

^¶^ *p*-values obtained from independent samples t- test.

^R^ *p*-values obtained from Mann-Whitney U test.

^*^P ≤ 0.05 was considered as statistically significant.

B.Sc., bachelor of science; M.Sc., master of science; BMI, body mass index; WC, waist circumference; BDI-II, Beck depression inventory-II test; MoCA, Montreal Cognitive Assessment tool; LPS, lipopolysaccharide; CRP, C-reactive protein; CFU, Colony Forming Unit.

**Table 2:** Dietary intake and physical activity levels at baseline and after 9 weeks' intervention.

**Variable**	**Group A** ^**a**^				**Group B** ^**b**^				***P*-value^e^**
	**Baseline**	**Post-intervention**	**Δ^c^**	***P*-value^d^**	**Baseline**	**Post-intervention**	**Δ^c^**	***P*-value^d^**	
Energy (kcal/d)	2029.24 ± 452.80	2120.24 ± 468.05	91.00 ± 429.63	0.460	1734.66 ± 390.95	1805.78 747.49	71.12 ± 797.34	0.744	0.937
Carbohydrate (g/d)	289.51 ± 98.72	313.33 ± 76.97	57.76 ± 160.95	0.223	247.71 ± 58.78	258.71 ± 89.25	54.40 ± 136.44	0.701	0.954
Protein (g/d)	72.78 ± 15.39	75.83 ± 22.35	3.05 ± 23.08	0.642	70.24 ± 25.22	73.43 ± 29.64	3.18 ± 43.16	0.787	0.992
Fat (g/d)	64.71 (49.71, 84.51)	65.89 (49.87, 83.25)	0.97 ± 25.91	0.917	55.60 (43.68, 71.18)	65.93 (39.69, 88.68)	12.46 59.35	0.397	0.526
SFA (g/d)	17.18 ± 6.68	16.04 ± 4.20	−1.13 ± 8.00	0.620	13.10 ± 3.60	16.29 ± 8.15	3.19 ± 7.80	0.150	0.168
MUFA (g/d)	17.95 ± 6.24	18.59 ± 5.78	0.64 ± 7.88	0.773	16.07 ± 3.77	18.26 ± 7.25	2.18 ± 6.78	0.249	0.589
PUFA (g/d)	23.48 ± 12.35	22.22 ± 10.33	−1.26 ± 15.72	0.777	19.99 ± 5.14	22.05 ± 13.97	2.06 ± 14.28	0.597	0.569
Magnesium (mg/d)	184.37 (157.59, 227.56)	167.85 (142.18, 226.11)	−3.54 (−62.69, 25.08)	0.507	163.19 (138.64, 222.65)	176.44 (135.19, 238.14)	14.18 (−61.95, 72.24)	0.778	0.192
Fiber (g/d)	12.84 ± 4.13	12.56 ± 3.26	0.45 ± 4.24	0.78	11.87 ± 5.46	13.70 ± 4.16	4.38 ± 11.77	0.787	0.267
Sugar (g/d)	42.55 (30.79, 60.31)	46.91 (35.77, 62.56)	−5.75 ± 21.63	0.507	37.64 (23.45, 58.94)	45.66 (38.46, 69.62)	9.51 ± 38.82	0.470	0.223
PA (METs-hr/d)	34.52 ± 4.55	31.68 ± 4.66	−2.84 ± 5.96	0.190	32.91 ± 4.57	31.12 ± 6.67	−1.79 ± 8.04	0.522	0.757

Data are presented as mean ± SD and median (Q1, Q3) for normally and non-normally distributed variables, respectively.

^a^Group A: intervention group, received one probiotic capsule (Probio-Tec^®^BG-VCap-6.5 Pla V2, containing 1.8 × 10^10^ CFU *Lactobacillus rhamnosus* and *Bifidobacterium animalis* subsp. *Lactis*) plus one magnesium chloride capsule (containing 125 mg elemental magnesium), on a daily basis for 9 weeks.

^b^Group B: received two placebo capsules containing maltodextrin on a daily basis for 9 weeks.

^C^Δcalculated as: (post-intervention – baseline) in each study group.

^d^*P*-value for within-group comparisons, obtained from paired samples *t*-Test and Wilcoxon signed ranked test for normally and non-normally distributed variables, respectively.

^e^*P*-value for between-group comparisons, obtained from independent samples *t*-Test and Mann-Whitney U test for normally and non-normally distributed variables, respectively.

^*^P ≤ 0.05 was considered as statistically significant.

SFA, saturated fatty acids; MUFA, monounsaturated fatty acids; PUFA, polyunsaturated fatty acids; PA, physical activity; METs, metabolic equivalents; CFU, Colony Forming Unit.

**Table 3:** Outcome variables at baseline and after 9 weeks' intervention.

**Variable**	**Group A** ^**a**^				**Group B** ^**b**^				***p*-value**
	**Baseline**	**Post-intervention**	**Δ^c^**	***P*-value**	**Baseline**	**Post-intervention**	**Δ^c^**	***p*-value**	
Serum Magnesium (mg/dl)	2.00 ± 0.16	1.97 ± 0.16	−0.03 ± 0.16	0.179^£^	1.97 ± 0.22	2.00 ± 0.23	0.03 ± 0.13	0.155^£^	0.053^**¶**^
Serum Zonulin (ng/ml)	13.73 (8.92, 18.88)	15.06 (9.63, 18.53)	0.11 ± 5.13	0.858^Ω^	13.02 (10.00, 18.25)	13.30 (10.48, 16.80)	−0.17 ± 6.60	0.455^Ω^	0.873^**¶**^
Serum LPS (ng/ml)	217 (179.50, 256.50)	210.50 (171.75, 262.25)	−3.04 ± 44.75	0.485^Ω^	217.00 (181.00, 264.00)	229 (178.75, 264.75)	1.40 ± 48.78	0.661^Ω^	0.754^**¶**^
CRP (mg/l)	5705.95 ± 3583.72	5231.19 ± 3576.39	−474.75 (−1300,−125)	0.090^£^	6201.82 ± 4705.63	6701.18 ± 4235.99	175.20 (−957.75, 1683.25)	0.240^£^	0.016^R^
BDI-II (score)	21.00 (15.00, 29.00)	15.00 (7.00, 22.00)	−7.13 ± 5.67	< 0.001^Ω^	20.50 (15.00, 25.00)	13 (8.00, 24.50)	−5.42 ± 6.71	< 0.001 ^Ω^	0.246^**¶**^
MoCA (score)	24.99 ± 2.99	26.12 ± 2.66	1.20 ± 2.16	0.001^£^	24.20 ± 3.47	26.15 ± 2.88	1.94 ± 1.86	< 0.001^£^	0.124^**¶**^

Data are presented as mean ± SD and median (Q1, Q3) for normally and non-normally distributed variables, respectively.

^a^Group A: intervention group, received one probiotic capsule (Probio-Tec^®^BG-VCap-6.5 Pla V2, containing 1.8 × 10 ^10^ CFU *Lactobacillus rhamnosus* and *Bifidobacterium animalis* subsp. *Lactis*) plus one magnesium chloride capsule (containing 125 mg elemental magnesium), on a daily basis for 9 weeks.

^b^Group B: received two placebo capsules containing maltodextrin on a daily basis for 9 weeks.

^c^ Δ calculated as: [post-intervention – baseline] in each study group.

^£^ *p*-values obtained from paired samples *t*-test.

^Ω^ *p*-values obtained from Wilcoxon signed ranked test.

^¶^ *p*-values obtained from independent samples t- test.

^R^ *p*-value obtained from Mann-Whitney U test.

^*^P ≤ 0.05 was considered as statistically significant.

BMI, body mass index; WC, waist circumference; Mg, Magnesium; CRP, C-reactive protein; LPS, lipopolysaccharide; MoCA, Montreal cognitive assessment test; BDI-II, Beck depression inventory-II test; CFU, Colony Forming Unit.

In order to adjust potential confounders, multiple linear regression model was conducted for evaluation of between group comparison. In this model, changes in outcome variable (post-intervention minus baseline) were entered as dependent variable while participants' BMI, education, job, income, BDI-II as well as energy and macronutrient intakes were regarded as covariates. Although after adjustment, no differences were observed in the study results.

## Discussion

### Effects of probiotic and magnesium co-supplementation on intestinal barrier function and systemic inflammation

Our study revealed that 9 weeks' supplementation with probiotic and magnesium in individuals with obesity and depression might improve CRP levels with no significant effects on serum zonulin and LPS concentrations as markers of intestinal integrity. Our findings are consistent with a previous study conducted by Lee et al. evaluating effects of herbal medicine with or without probiotics on gut microbiota, gut permeability and endotoxin levels in subjects with overweight/obesity. Similar to our results, no significant changes were observed in LPS, intestinal barrier function and other metabolic markers (62). In a 12-week trial of post-menopausal women with obesity, it was shown that probiotic supplementation might beneficially affect LPS levels in a dose-response manner (63). In another 4-month clinical trial carried out on individuals undergoing gastric bypass surgery, multispecies probiotic could improve levels of LPS binding protein, TNF-α and weight loss (64). Amirani et al. conducted a metaanalysis on the effects of probiotics on inflammatory markers in participants with psychiatric disorders. A significant reduction in CRP and Interleukine-10 (IL-10) levels was seen following probiotic consumption (65). Exact mechanisms through which probiotics exert beneficial effects on inflammation and gut barrier function are not completely elucidated. Probiotic strains have the potential to enhance epithelial barrier integrity through modulating gene expression of adhesion proteins (47, 66) and production of health promoting molecules and anti-microbial peptides which prevent pathogen growth (67, 68). Probiotics also modulate host immune system by microbe-associated molecular patterns (MAMPs) which interact with pattern recognition receptors (PRRs) present on the surface of intestinal epithelial and immune cells and maintain immune homeostasis (69, 70) which might not only improve intestinal barrier integrity, but also play a role in regulation of inflammatory state (70). Probiotics produce surface-layer proteins (SLPs) that reduce LPS induced inflammation through decreased translocation of NF-Kβ into nucleus which eventually attenuates TNF-α, IL-1β and oxidative stress (70).

Magnesium deficiency is associated with an inflammatory state characterized by elevated levels of acute phase proteins (71). The inverse relationship between magnesium intake and inflammatory state have been reported in several studies as reviewed by Belin and He (72). However, studies regarding direct effects of magnesium intake on intestinal integrity and gut microbiota are rare. Mice fed with magnesium deficient diet had a lower gut *Bifidobacteria* content, lower mRNA levels encoding factors involved with intestinal barrier integrity (zonula-occludens-1, occluding, proglucagon), increased expression of TNF-α, IL-6 and activating transcription factor-4, a reflection of inflammatory and cellular stress (39).

Based on previous studies, a longer study duration might be needed to observe potential improvements in gut barrier function while inflammatory markers such as CRP levels take less time to be influenced by probiotics or dietary supplements. Furthermore, since our intervention contained magnesium, inflammatory status might be improved by pathways related to weight reduction which occurred in our intervention group; After analyzing anthropometric findings, we realized that participants in intervention group had considerable reductions in weight (−4.99 ± 1.32 kg, *p* = 0.012), BMI (−1.95 ± 0.51 kg/m^2^, *p* = 0.012) and WC (−1.58 ± 1.51 cm, *p* < 0.001). Therefore, reduced adiposity might be a potential explanation for at least part of inflammation improvement in our study.

### Effects of probiotic and magnesium co-supplementation on mood and cognition

BDI-II and MoCA as indicators of mood and cognitive performance improved in both groups with no significant between-group differences. This finding can be justified by a couple of logics; As stated by evidence, BDI-II is a standardized self-report measure to identify depressive disorders and categorize the severity of depressive symptoms (73). We assume that subjective nature of this tool and placebo effect might be the reason for significant improvements in control group. Actually a recent 2022 publication has clearly stated that “depression is a highly placebo responsive condition” (74).

Regarding cognitive assessment, MoCA is an excellent and simple tool which evaluates multiple cognitive domains with great sensitivity and specificity for detecting mild cognitive impairment (MCI) (75). However, since participants performed MoCA before and after 9 weeks, and regarding the fact that several sections of MoCA test are memory based, elevated MoCA scores in control group might be due to participants' task learning and memorization.

Several clinical trials have been conducted in this area with various results. In a 12-week randomized clinical trial, Akbari et al. demonstrated that a mixture of probiotics can significantly improve cognition evaluated by Mini- mental state examination (MMSE) in patients with Alzhimer's disease (AD) (76). Furthermore, a metaanalysis of four randomized trials, revealed beneficial effects of probiotic supplementation in Hamilton Depression Rating Scale (HAMD) (65). Probiotic supplementation along with magnesium was carried out in one small pilot study. Eight weeks' consumption of probiotics and magnesium orotate significantly improved depression scores and quality of life in 12 participants with drug resistant depression (77).

Probiotics can exert promoting effects on mood and cognition through several mechanisms. Since persistent low grade inflammation is associated with existence and severity of depressive symptoms, probiotics might relieve such symptoms *via* anti-inflammatory activities explained earlier in this section (78). Furthermore, gut microbiota and probiotics are known to synthetize neurotransmitters responsible for maintaining proper brain function including gamma amino butyric acid (GABA), serotonin (5-HT), glutamate (Glu), dopamine and norepinephrine (NE). Gut microbiota also regulates the bioavailability of precursors for these neurotransmitters (79). Besides these mechanisms, research has indicated that gut microbiota elicits signals to the brain *via* vagus nerve and vice versa (80).

Magnesium has long been used to treat depression and relieve a variety of emotional problems even in homeopathic medicine (81). Studies in this area has been going on so far. Tarleton et al. showed that 6 weeks consumption of magnesium chloride can improve depressive symptoms (82). In another study, consumption of 500 mg magnesium oxide for 8 weeks led to improvements in depressive symptoms and serum magnesium levels in participants with depression and hypomagnesemia (83). In the present study, serum magnesium levels did not change significantly following magnesium chloride consumption. It might be due to our finding that mean baseline serum magnesium levels of participants was 1.99 ± 0.19 mg/dl, which already drops within normal range (84).

The role of magnesium in intestinal barrier function remains to be elucidated. In a study by Pachikian et al., 4 days of magnesium deficiency resulted in decreased ileal expression of Occludins, Zo-1 and Zo-2 in mice. Magnesium deficient mice also exhibited discontinuous Zo-1 and Occludin staining in the ileum compared with control group (39). In addition to the crucial role of magnesium in ATP metabolism which is essential for normal neurological function and neurotransmission (85), it has been regarded as one of the modulators of N-methyl-D-aspartate (NMDA), a receptor complex involved in pathophysiology of depression and is considered as a target for anti-depressant therapy (86).

Our study has some limitations. Although we started our study with an adequate sample size, our dropout rate was a bit higher than our expectation which was partly due to COVID-19 pandemic. Nevertheless, despite dropouts, an acceptable number of participants remained in each group and for missing data, imputation method was carried out. We had to decrease daily dose of magnesium chloride from two to one 500 mg capsule per day for better compliance, which might have attenuated probable effects of magnesium on study outcomes. Markers such as IL-6 and TNF-α could be measured besides CRP as parameters of inflammatory status but due to financial limitations we did not include such parameters as outcomes. Although BDI-II and MoCA tools have high reliability and validity due to their subjective nature they might have been affected by different conditions.

In spite of these limitations our study has several strengths. To the best of our knowledge, this was the first study which has evaluated effects of a combination of probiotic and magnesium on markers of intestinal integrity, mood, cognition and serum CRP levels in individuals with obesity and depressed mood. We did our best to control as many potential confounders as possible to increase the validity of our findings. Probiotics used in this study were Probio-Tec^®^BG-VCap-6.5 with accurate product information and analysis manufactured by Chr. Hansen according to food laws and legislations. For magnesium, we used its chloride salt which has a higher bioavailability and tolerability than other magnesium salts (87).

Overall, 9 weeks of probiotic and magnesium supplementation resulted in decreased CRP levels in individuals with obesity and depressed mood. However, this intervention was ineffective in improving intestinal barrier function, mood and cognition. It is suggested that future research in this area consider longer durations, higher doses of magnesium and apply objective tools for neurocognitive assessments.

## Data availability statement

The raw data supporting the conclusions of this article will be made available by the authors, without undue reservation.

## Ethics statement

The study protocol was drafted and conducted according to Declaration of Helsinki and CONSORT statements, and approved by the Ethics Committee of Shiraz University of Medical Sciences (approval code: IR.SUMS.REC.1398.1375). All participants provided written informed consent prior to the study commencement.

## Author contributions

SM and ME contributed in research conceptualization. SM, ME, and HG developed study design, methodology, validation, and took part in the manuscript finalization and improvement. SM and MG participated in investigation process, experiments, data collection, data entry, drafted the original manuscript, and contributed to data visualization. HG and SM contributed in data analysis and curation. SA contributed in providing study resources. All study phases were conducted with the supervision of ME. All authors contributed to the article and approved the submitted version.

## Funding

This manuscript was extracted from a PhD dissertation funded by Vice President of Research and Technology (Research Code: 20052-84-01-98), Shiraz University of Medical Sciences, Shiraz, Iran.

## Conflict of interest

The authors declare that the research was conducted in the absence of any commercial or financial relationships that could be construed as a potential conflict of interest.

## Publisher's note

All claims expressed in this article are solely those of the authors and do not necessarily represent those of their affiliated organizations, or those of the publisher, the editors and the reviewers. Any product that may be evaluated in this article, or claim that may be made by its manufacturer, is not guaranteed or endorsed by the publisher.
